# Characterization of the roles of amphiregulin and transforming growth factor β1 in microvasculature-like formation in human granulosa-lutein cells

**DOI:** 10.3389/fcell.2022.968166

**Published:** 2022-08-24

**Authors:** Hui Li, Hsun-Ming Chang, Saijiao Li, Christian Klausen, Zhendan Shi, Peter C.K. Leung

**Affiliations:** ^1^ Jiangsu Key Laboratory for Food Quality and Safety-State Key Laboratory Cultivation Base of Ministry of Science and Technology, Key Laboratory of Animal Breeding and Reproduction, Institute of Animal Science, Jiangsu Academy of Agricultural Sciences, Nanjing, China; ^2^ Department of Obstetrics and Gynaecology, BC Children’s Hospital Research Institute, University of British Columbia, Vancouver, BC, Canada; ^3^ Reproductive Medicine Center, Department of Obstetrics and Gynecology, China Medical University Hospital, Taichung, Taiwan; ^4^ Reproductive Medical Center, Renmin Hospital of Wuhan University, Wuhan, China

**Keywords:** VE-cadherin, TGF-β1, amphiregulin, microvasculature-like formation, human granulosa cells

## Abstract

Vascular endothelial-cadherin (VE-cadherin) is an essential component that regulates angiogenesis during corpus luteum formation. Amphiregulin (AREG) and transforming growth factor β1 (TGF-β1) are two intrafollicular factors that possess opposite functions in directing corpus luteum development and progesterone synthesis in human granulosa-lutein (hGL) cells. However, whether AREG or TGF-β1 regulates the VE-cadherin expression and subsequent angiogenesis in the human corpus luteum remains to be elucidated. Results showed that hGL cells cultured on Matrigel spontaneously formed capillary-like and sprout-like microvascular networks. Results of specific inhibitor treatment and small interfering RNA-mediated knockdown revealed that AREG promoteed microvascular-like formation in hGL cells by upregulating the VE-cadherin expression mediated by the epidermal growth factor receptor (EGFR)-extracellular signal-regulated kinase1/2 (ERK1/2) signaling pathway. However, TGF-β1 suppressed microvascular-like formation in hGL cells by downregulating VE-cadherin expression mediated by the activin receptor-like kinase (ALK)5-Sma- and Mad-related protein (SMAD)2/3/4 signaling pathway. Collectively, this study provides important insights into the underlying molecular mechanisms by which TGF-β1 and AREG differentially regulate corpus luteum formation in human ovaries.

## Introduction

Angiogenesis is crucial in follicular development, corpus luteum formation, and progesterone secretion (Suzuki et al., 1998; Sugino et al., 2008). This process actively occurs from the early luteal to the mid‐luteal phase (Sugino et al., 2008). Before ovulation, blood vessels are restricted to the theca cell (TC) layer, and extensive physiological vascularization invades the avascular granulosa cell (GC) layer during corpus luteum formation (Suzuki et al., 1998; Gruemmer et al., 2005). Within the neovascular network, substrates for progesterone production, energy supply, hormones, oxygen, endocrine factors, and metabolic waste can be transported bidirectionally (Nakhuda et al., 2005). Previous studies demonstrate that the development of the corpus luteum is disturbed and serum progesterone is significantly reduced when the process of neovascularization is inhibited (Nakhuda et al., 2005). Moreover, clinical studies revealed that aberrant angiogenic processes in ovarian follicles after administration of a high dose of gonadotropins may increase the risk of developing ovarian hyperstimulation syndrome (OHSS) (Agrawal et al., 1998). Taken together, these findings suggest that follicular neovascularization is tightly controlled and essential in corpus luteum formation.

A number of studies have shown that follicular GCs possess functions similar to endothelial-like cells, because GCs not only express endothelial cell markers but also participate in microvascular and network formation (the typical characteristics of endothelial cells) (Antczak and Van Blerkom, 2000; Li et al., 2021). Thus, GCs possibly have the potential to participate in vascularization during the corpus luteum development. However, the detailed functions of GCs in vascularization are unclear. Vascular endothelial-cadherin (VE-cadherin, other name is CD144), which belongs to the cadherin superfamily and is encoded by the cadherin 5 (CDH5) gene (Suzuki et al., 1991). VE-cadherin is an endothelial transmembrane protein localized in intercellular junctions and crucial in maintaining vascular integrity and neovascularization (Vestweber, 2008). It is also detected in the GCs and TCs of the human corpus luteum (Groten et al., 2006). An *in vitro* animal model study showed that downregulation of VE-cadherin expression disrupts capillary-like network formation in endothelial cells derived from the bovine corpus luteum (Maroni and Davis, 2011). Additionally, *in vivo* administration of an antibody against VE-cadherin blocks corpus luteum formation and angiogenesis in mice (Nakhuda et al., 2005). Collectively, these findings indicate that VE-cadherin is essential in angiogenesis during corpus luteum formation.

Amphiregulin (AREG) belongs to the epidermal growth factor-like (EGF-like) family. Members of this family share a highly similar structure (Schneider and Wolf, 2009; Richani and Gilchrist, 2018), and exert their similar biofunctions by binding to four specific receptors (EGFR, HER2, HER3, and HER4) in an autocrine or paracrine manner (Borrell-Pages et al., 2003; Bublil and Yarden, 2007). Recent studies have shown that EGF-like growth factors (especially AREG) act as secondary propagators produced by GCs in response to luteinizing hormone (LH) (Su et al., 2002; Richani and Gilchrist, 2018). Additionally, their functional receptor EGFR is highly expressed in ovarian follicles during the preovulatory and luteal phases (Akayama et al., 2005; Richani and Gilchrist, 2018). Indeed, studies have shown that AREG promotes progesterone production (Ben-Ami et al., 2009) and increases the expression of vascular endothelial growth factor (VEGF, an important factor that induces angiogenesis) in human GCs (Fang et al., 2019). These results suggest that AREG regulates angiogenesis during corpus luteum development.

Transforming growth factor beta1 (TGF-β1) and its receptors are detected in the GCs and TCs of large ovarian follicles in humans (Chegini and Flanders, 1992; Rosairo et al., 2008; Kristensen et al., 2014). This multifunctional growth factor exerts various cellular activities *via* type II and type I receptor (TβRI, also named as ALK5). The ligand-receptor complex subsequently regulates target gene expression by activating the downstream mediators Sma- and Mad-related proteins (SMADs), especially SMAD2/3 and SMAD4 (the common SMAD) (Annes et al., 2003). Studies performed by our laboratory and others have illustrated that TGF-β1 suppresses luteinization and promotes luteolysis by suppressing the expression of steroidogenic acute regulatory protein (StAR) and decreasing the secretion of progesterone (Miyamoto et al., 1992; Hou et al., 2008; Fang et al., 2014). Additionally, it alsoinhibits luteinization by suppressing the expression of luteinization-related genes (such as MMP1) in mammals and humans (Zheng et al., 2009; Li et al., 2019). Moreover, the administration of a luteolytic dose of prostaglandin F2 induces TGF-β1 expression in the corpus luteum of rats, mice and bovines (Stocco et al., 2001; Wang et al., 2003; Hou et al., 2008). Indeed, our most recent studies also showed that TGF-β1 prevents microvascular-like network in hGL cells by down-regulating VCAM1 and ICAM1 (two key molecules of intercellular junction) (Li et al., 2021). In view of this, TGF-β1 may affect corpus luteum development by suppressing vascularization in human ovarian follicles.

These results suggest that follicular vascularization and the corpus luteum development are tightly controlled by locally produced growth factors. Therefore, we hypothesized that three intraovarian factors, AREG, TGF-β1 and VE-cadherin, play differential roles in regulating these physiological processes. To prove this hypothesis, we characterized the roles of AREG and TGF-β1 in regulating microvasculature-like network formation and their regulatory mechanisms in cultured human granulosa-lutein (hGL) cells.

## Materials and methods

### Human granulosa-lutein cell line (SVOG cell) and primary human GL cells culture

An immortalized cell line (SVOG cell) (Chang et al., 2016a) was used in this study. Primary hGL cells were used to generate our immortalized SVOG cells, and the 2 cell types display similar biological responses (express similar levels of receptors and induce the same intracellular signaling) to many different growth factor treatments. SVOG cells were cultured in DMEM/F-12 supplemented with 10% fetal bovine serum (HyClone, UT), penicillin (100 U/mL), streptomycin sulfate (100 μg/ml) and 1× GlutaMAX (Invitrogen, NY) in 5% CO_2_ at 37°C. Cells need serum starvation 24 h before further treatment.

Following the approval of Research Ethics Board (the University of British Columbia), the follicle fluid collected from 16 anonymized women undergoing oocyte retrieval were transferred to lab in 2 h after collection. The hGL cells were isolated as described by Chang (Chang et al., 2013; Chang et al., 2016b).

### Antibodies and reagents

All the primary antibodies including anti-VE-cadherin, anti-p-EGFR, anti-p-ERK1/2 anti-HER2 antibodies were produced by Cell Signaling Technology (Beverly, MA). Anti-α-tubulin (sc-23948), HRP- or Alexa Fluor 594- conjugated second antibodies were purchased from Santa Cruz Biotechnology. Recombinant proteins, including human AREG and TGF-β1, inhibitors: AG1478 and U0126 were produced by R&D Systems (Minneapolis, MN). Matrigel was purchased from BD Biosciences (Franklin Lakes, NJ).

### RT-qPCR analysis

TRIzol reagent (Invitrogen, United States) was used to extract cellular total RNA. cDNA was generated using Moloney murine leukemia virus (MMLV) reverse transcriptase (Promega, Madison), and subjected to qPCR using 2× SYBR Green PCR Mix (Applied Biosystems, United States). Each reaction contained, 100 ng of cDNA. The primers used for PCR reaction are shown in Table 1. RT-qPCR was performed on ABI 7300 System. The mRNA expression levels were calculated using the comparative Ct (2^–∆∆Ct^) method, and GAPDH was used as the house keeping gene. Each sample was assayed three times.

**TABLE 1:** Primers used in this study.

Gene	Primer sequences (5′–3′)	Length (bp)
GAPDH	F: GAG​TCA​ACG​GAT​TTG​GTC​GT	185
	R: GACAAGCTTCC CGTTCTCAG	
VE-cadherin	F: GCG​ACT​ACC​AGG​ACG​CTT​TCA	149
	R: CAT​GTA​TCG​GAG​GTC​GAT​GGT​G	
EGFR	F: GGT​GCA​GGA​GAG​GAG​AAC​TGC	270
	R: GGT​GGC​ACC​AAA​GCT​GTA​TT	
HER2	F: AAC​TGC​ACC​CAC​TCC​TGT​GT	142
	R: TGA​TGA​GGA​TCC​CAA​AGA​CC	
HER3	F: CAA​GAT​TCC​AGT​CTG​CAT​TAA​AGT​C	79
	R: CAG​CAT​ATG​ATC​TGT​CAC​AGC​TTG	
HER4	F: CAA​CAT​CCC​ACC​TCC​CAT​CTA​TAC	149
	R: ACA​CTC​CTT​GTT​CAG​CAG​CAA​A	

### Western blot

Total proteins were extracted using lysis buffer (Cell Signaling Tech) supplemented with 1× protease or phosphatase inhibitor (Transgene). The protein concentrations were subsequently quantified, and 30 μg protein in each sample was loaded and electrophoretic separated by 8% or 12% SDS-PAGE and then transferred onto the polyvinylidene fluoride (PVDF) membranes. The membranes were subsequently incubated overnight at 4°C with the primary antibodies (diluted at 1:1,000) after blocking by 5% BSA. Then, they were thoroughly washed with TBST and incubated with their relevant HRP-conjugated antibodies. Protein bands were exposed to X-ray film (Thermo Fisher, United States) using the SuperSignal kit (TransGen Biotech). Protein band intensity was quantified using ImageJ (Bethesda Maryland), and three separated repeats were conducted.

### siRNA transfection

Nontargeting control or siRNA targeting EGFR, ERK1/2, ALK4, ALK5, SMAD2, SMAD3, SMAD4 and VE-cadherin (siEGFR: L-003114-00-0005; siERK1/2: L-005028-00-0005; siALK4: L-004925-00-0005; siALK5: L-003929-00-0005; siSMAD2: L-003561-00-0005; siSMAD3: L-020067-00-0005; siSMAD4: L-003902-00-0005; and siVE-cadherin: L-003641-00-0005) (Dharmacon, GE Health Care) were used to perform transient knockdown assays. 25 nM siRNA were transfected into the cells (at 50% confluence) using Lipofectamine RNAi MAX (Life Technologies) as previously described (Wu et al., 2017; Bai et al., 2018).

### Immunofluorescent staining

Cells were pre-seeded on laminin-coated 8-well slides (Corning). After being treated with AREG, TGF-β1, inhibitors or siRNA-mediated knockdown, the cells were fixed and incubated with rabbit anti-VE-cadherin antibody (diluted at 1:500) overnight at 4°C. The cells were then thoroughly washed and incubated with Alexa Fluor 594-conjugated goat anti-rabbit IgG (Abcam) for further 2 h at 4°C. After thoroughly washing again, the nuclei were stained with DAPI solution (1 μg/ml) for 5 min. Cell slides were maintained in the dark until images were taken under a fluorescence microscope. The fluorescence intensity in each field in the slides was quantified using ImageJ (Bethesda Maryland).

### Microvascular (capillary) network and sprouting formation assay

Firstly, 96-well plates used for microvascular (capillary) network and sprouting formation assay were precoated with Matrigel (BD, Biosciences) as previously described by Li (Li et al., 2015; Li et al., 2021). The cells were pretreated with growth factors, inhibitors, or siRNA-mediated knockdown for 24 h; and transferred onto Matrigel-precoated 96-well plates (BD, Biosciences) (1 × 10^5^/well); and then cultured at 37°C in 5% CO_2_ for further 5 and 24 h (for microvascular network and sprouting formation assay, respectively). Images were taken under an inverted microscope. The capillary and sprout lengths were measured using an angiogenesis analyzer (ImageJ) as previously described (Li et al., 2021).

### Statistical analysis

Data statistics were performed by PRISM software (GraphPad Software, CA) using one-way ANOVA. For the normality test, we used the Shapiro-Wilk test as our numerical means of assessing normality, and the Sig. value of the Shapiro-Wilk Test is greater than 0.05, indicating that the data is normal. All the experiments were performed at least three separate repeats and the results are presented as the mean ± SEM. *p* < 0.05 was considered as significantly different.

## Results

### EGF receptors are detected in primary and immortalized human granulosa-lutein cells

**FIGURE 1:**
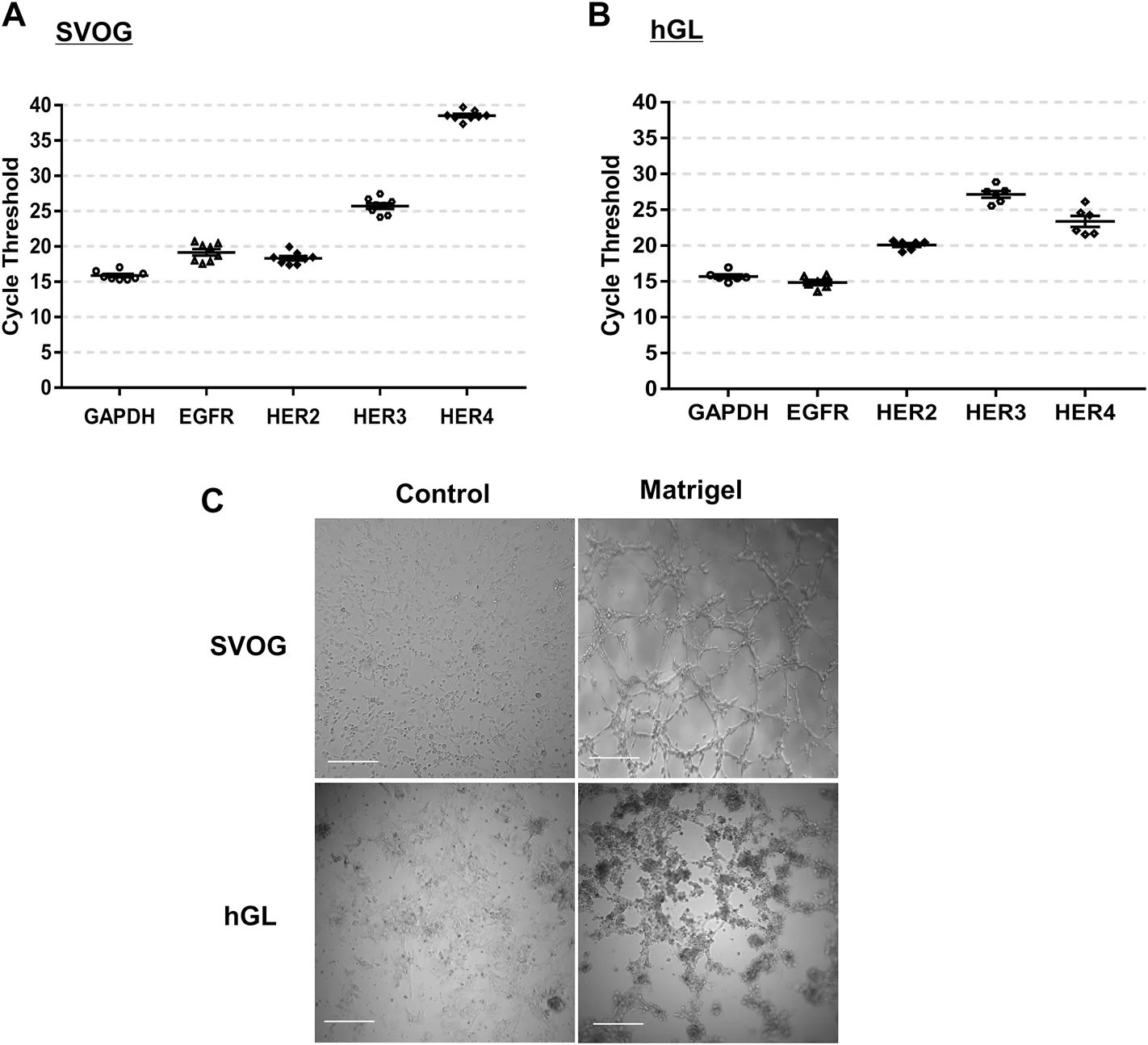
Expression of EGF-like growth factor receptors and microvasculature-like network formation in hGL cells. **(A,B)** GAPDH, EGFR, HER2, HER3 and HER4 expressions in SVOG [**(A)**, *n* = 8] and primary hGL cells [**(B)**, *n* = 6] were assayed by RT-qPCR. The distribution of the cycle threshold values is presented after the RT-qPCR. **(C)** In the microvascular-like formation assay, SVOG and primary hGL cells were cultured on non-Matrigel- and Matrigel-precoated plates for 5 h. Images were taken by a microscope. Scale bars:100 µm.

Given the important roles of EGF-like growth factors in regulating luteinization and corpus luteum development, we first examined the expression of their functional receptors in primary hGL and SVOG cells. As shown in Figures 1A,B, three EGF receptors (including EGFR, HER2 and HER3) were highly expressed in primary hGL and SVOG cells. The expression of HER4 was low in SVOG cells (Figure 1A), but high in primary hGL cells (Figure 1B).

### Culture of primary and immortalized human granulosa-lutein cells on matrigel results in the spontaneous formation of a microvascular-like network

Compared to the cells cultured on regular culture plates, cords- and microvascular-like network were spontaneously organized in the SVOG and primary hGL cells cultured on Matrigel for 5 h (Figure 1C). These characteristics are very similar to those of vascular endothelial cells (Wustehube et al., 2010; Maroni and Davis, 2011).

### Amphiregulin promotes but transforming growth factor β1 suppresses microvascular-like formation in SVOG cells

**FIGURE 2:**
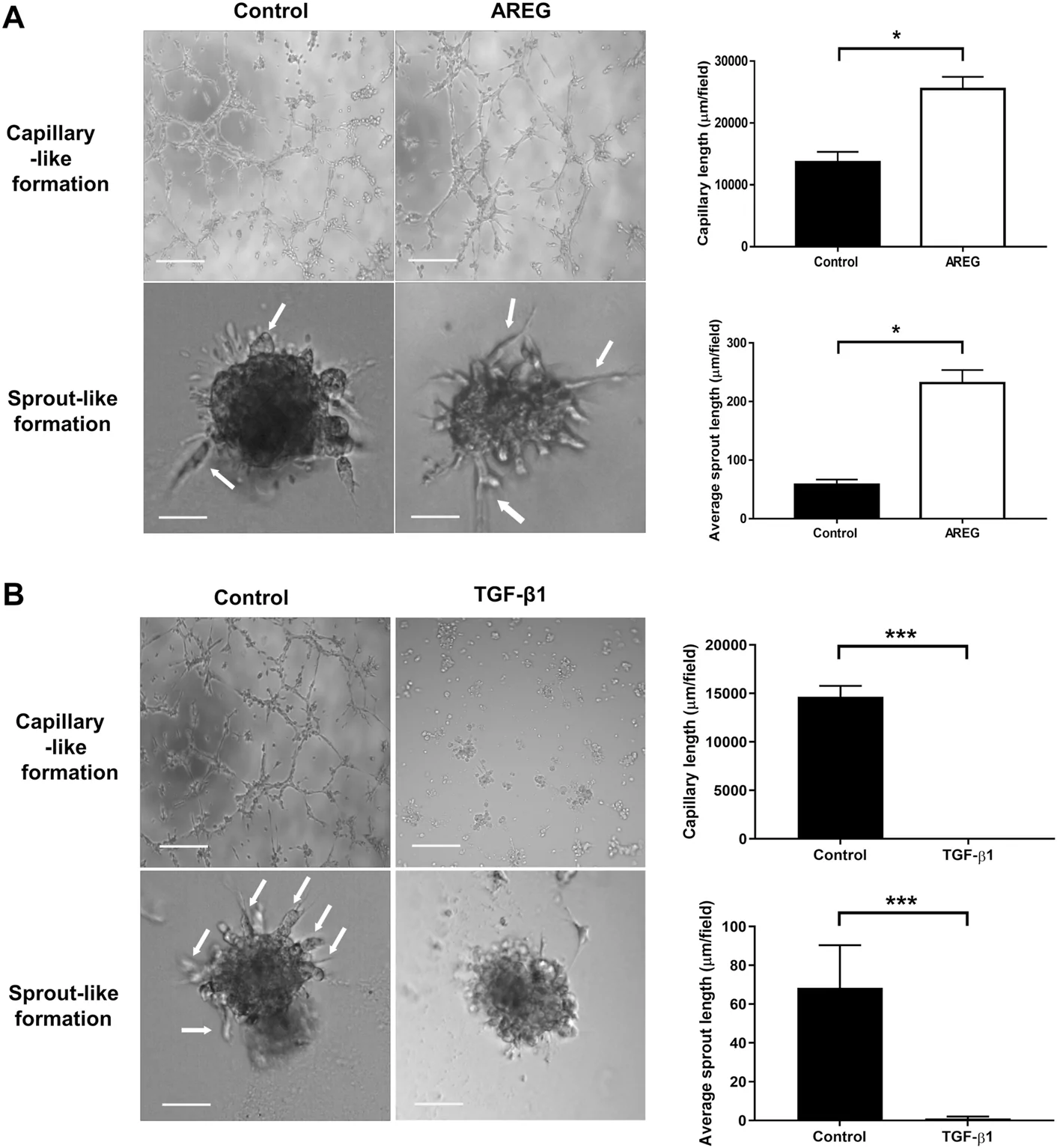
Effects of AREG and TGF-β1 on microvasculature-like formation in SVOG cells. Cells were pretreated with a vehicle control, AREG (100 ng/ml) **(A)** or TGF-β1 (10 ng/ml) **(B)** for 24 h. The cells were transferred to Matrigel for further 5 h (for capillary-like formation) and 24 h (for sprout-like formation). Scale bars of the capillary-like formation and sprout-like formation represent 100 µm and 50 μm, respectively. The average lengths of the capillaries and sprouts were quantified by ImageJ. Values labeled with different letters are significantly different (*p* < 0.05), *n* = 3.

We next characterized the functional roles of AREG and TGF-β1 in regulating microvascular-like formation in hGL cells. SVOG cells were pretreated with AREG (100 ng/ml) for 24 h and then transferred onto Matrigel-precoated 96-well plates for additional time points. Interestingly, the cells cultured on Matrigel for 5 h formed capillary-like formations, while cells cultured on Matrigel for 24 h formed sprout-like formations in response to AREG (Figure 2A). However, TGF-β1 (10 ng/ml) significantly suppressed both formations in SVOG cells (Figure 2B).

### Amphiregulin upregulates but transforming growth factor β1 downregulates VE-cadherin expression in SVOG cells

**FIGURE 3:**
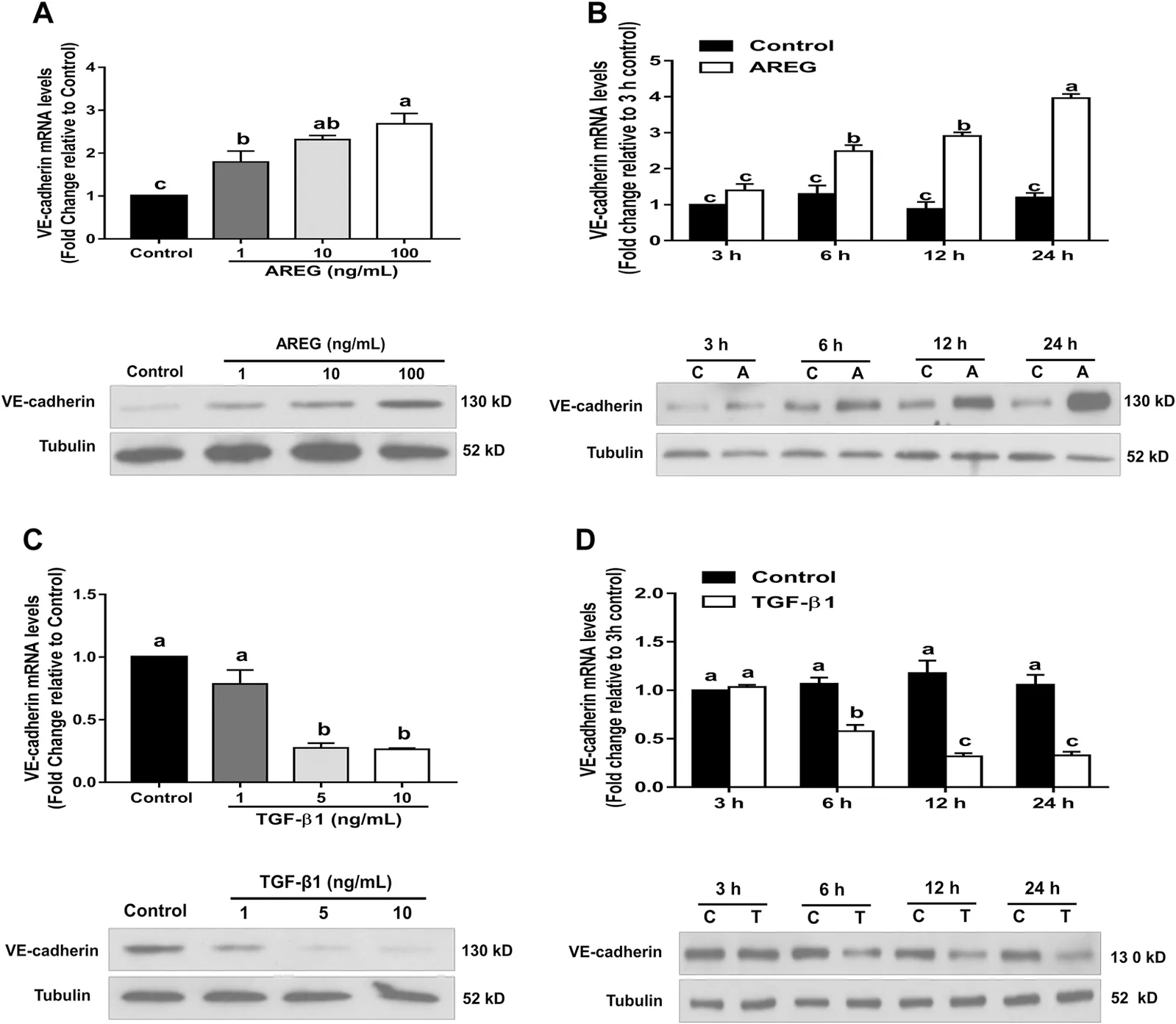
Effects of AREG and TGF-β1 on VE-cadherin expression in SOVG cells. **(A,C)** SVOG cells were treated for 24 h with or without varying concentrations of AREG (1, 10 or 100 ng/ml) **(A)** or TGF-β1 (1, five or 10 ng/ml) **(C)**, and the expression levels of VE-cadherin were examined using RT-qPCR and western blotting, respectively. **(B,D)** SVOG cells were treated with or without AREG (100 ng/ml) **(B)** or TGF-β1 (10 ng/ml) **(D)** for 3, 6, 12 or 24 h, and the expression levels of VE-cadherin were assayed by RT-qPCR and western blotting, respectively. Values labeled with different letters are significantly different (*p* < 0.05), *n* = 3. C, control; A, AREG; T, TGF-β1.

Considering that VE-cadherin engages in angiogenesis during corpus luteum formation (Richani and Gilchrist, 2018), we proposed that AREG and TGF-β1 control microvascular formation by differentially regulating VE-cadherin expression in hGL cells. To reveal it, different doses of AREG were used to treat SVOG cells. As shown in Figure 3A, AREG (for 24 h) increased the VE-cadherin expressions (both at the mRNA and protein levels). Time-course experiments were also performed, and the results exhibited that 100 ng/ml AREG (for 3, 6, 12 or 24 h) significantly upregulated VE-cadherin expression in SVOG cells (Figure 3B). By contrast, VE-cadherin expressions were downregulated in response to TGF-β1 treatment in SVOG cells in a dose- (1, five or 10 ng/ml) (Figure 3C) and time-dependent (for 3, 6, 12 or 24 h) manner (Figure 3D).

### The epidermal growth factor receptor-ERK1/2 signaling pathway mediates amphiregulin-induced VE-cadherin upregulation in SVOG cells

**FIGURE 4:**
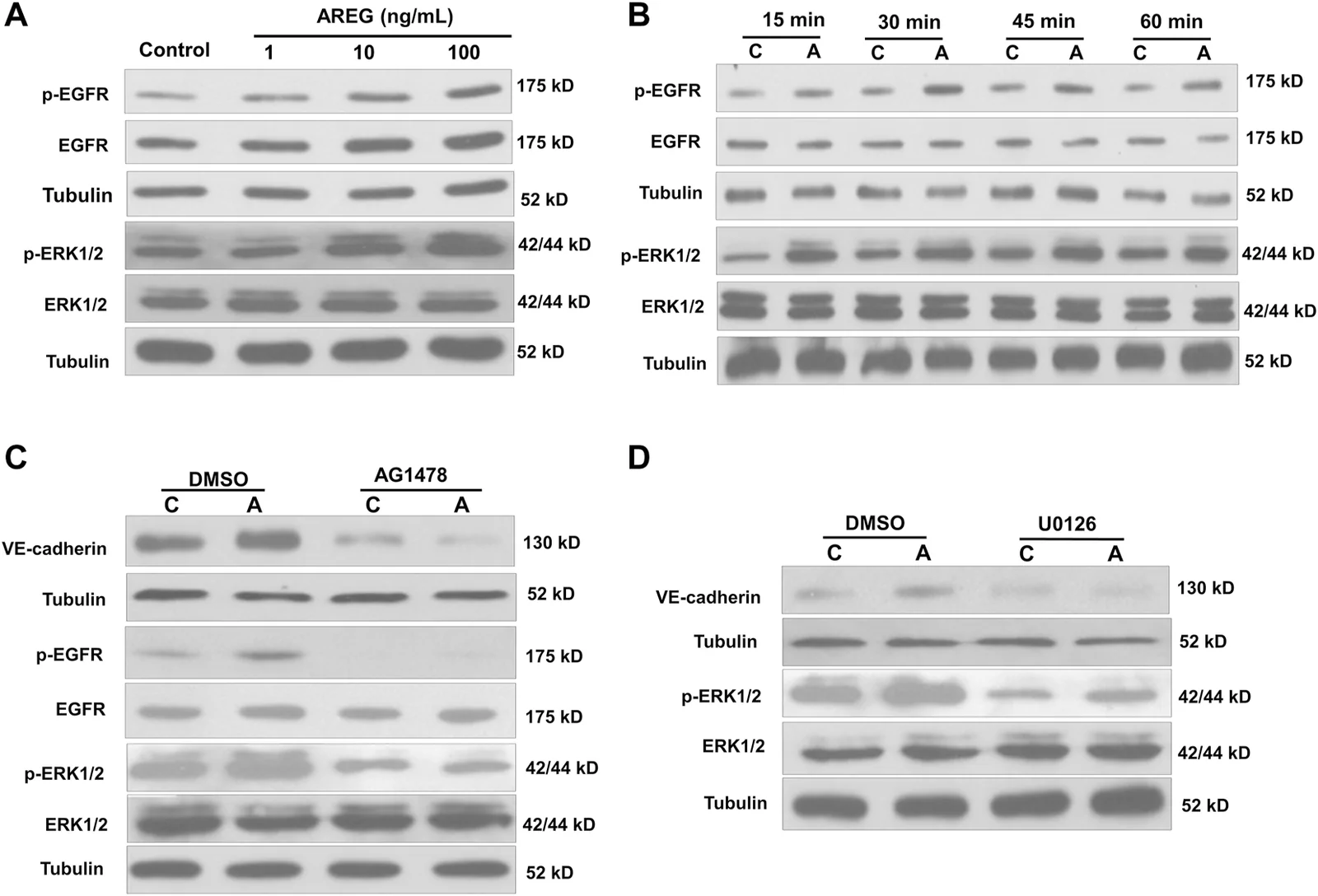
Effects of the inhibitors AG1478 and U0126 on the AREG-induced increase in phosphorylated EGFR and ERK1/2 in SVOG cells. **(A)** Cells were treated for 15 min with varying concentrations (0, 1, 10 or 100 ng/ml) of AREG, and the phosphorylation levels of EGFR and ERK1/2 were examined using western blotting. **(B)** Cells were treated with AREG (100 ng/ml) for 15, 30, 45 or 60 min, and the phosphorylation levels of EGFR and ERK1/2 were examined by western blotting. **(C,D)** SVOG cells were pretreated for 1 h with a vehicle control (DMSO), AG1478 (10 µM) **(C)** or U0126 (10 µM) **(D)** and then treated for an additional 15 min (for detecting phosphorylated EGFR) or 24 h (for detecting VE-cadherin) with or without AREG (100 ng/ml). The phosphorylation levels of EGFR, and VE-cadherin were examined by western blotting. Values labeled with different letters are significantly different (*p* < 0.05), *n* = 4. C, control; A, AREG.

**FIGURE 5:**
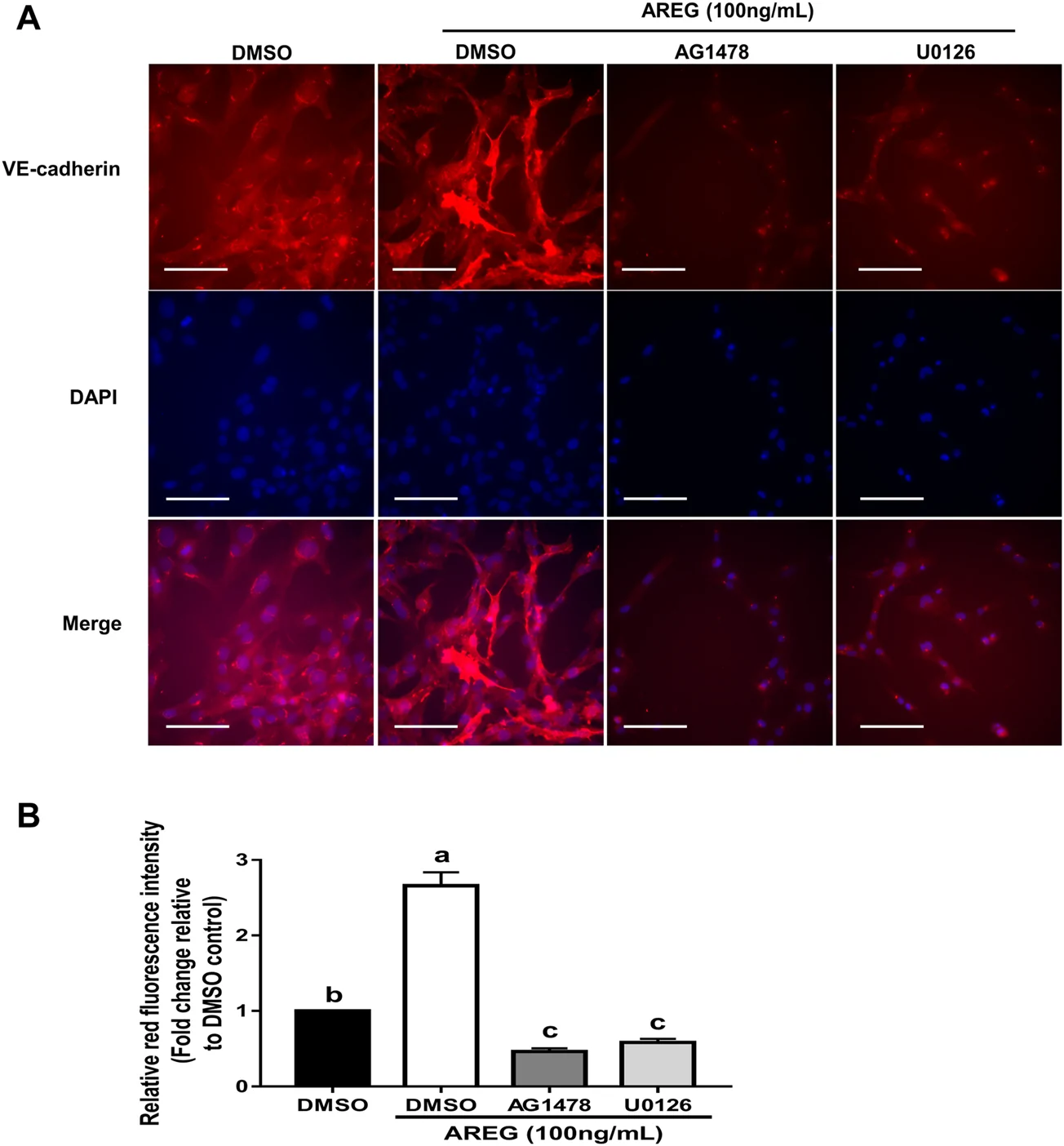
Fluorescence intensity assay showing the effects of the inhibitors AG1478 and U0126 on the AREG-induced upregulation of VE-cadherin in SVOG cells. **(A)** Cells were pretreated with DMSO, 10 µM AG1478 or 10 µM U0126 for 1 h and then treated for further 24 h with or without AREG (100 ng/ml). The VE-cadherin levels were determined using immunofluorescent staining **(B)**, and the fluorescence intensity was further quantified by ImageJ. Scale bars represent 100 µm. Values labeled with different letters are significantly different (*p* < 0.05), *n* = 3.

To reveal the molecular mechanism by which AREG regulates VE-cadherin expression, we examined the functional receptor and downstream signaling in response to AREG treatment in hGL cells. Results showed that AREG (1, 10 or 100 ng/ml for 15 min) increased the phosphorylation levels of EGFR and ERK1/2 with increasing concentration in SVOG cells (Figure 4A). Additionally, results of time-course studies showed that 100 ng/ml AREG time-dependently (for 15, 30, 45 or 60 min) increased the phosphorylation levels of EGFR and ERK1/2 (Figure 4B). Then, we used two kinase inhibitors, AG1478 and U0126, which can block the phosphorylation of EGFR and ERK1/2, respectively. Results showed that AG1478 treatment (for 1 h) completely reversed AREG-induced EGFR and ERK1/2 phosphorylation (Figure 4C). Additionally, pretreatment with U0126 (for 1 h) completely reversed AREG-induced ERK1/2 phosphorylation (Figure 4D). Moreover, pretreatment with either AG1478 or U0126 (for 1 h) completely abolished AREG-induced upregulation of VE-cadherin expression (Figures 4C,D). Indeed, fluorescent staining experiments using an antibody against VE-cadherin showed that pretreatment with either AG1478 or U0126 (for 1 h) significantly decreased AREG-induced increase in fluorescence intensity in SVOG cells (Figures 5A,B).

**FIGURE 6:**
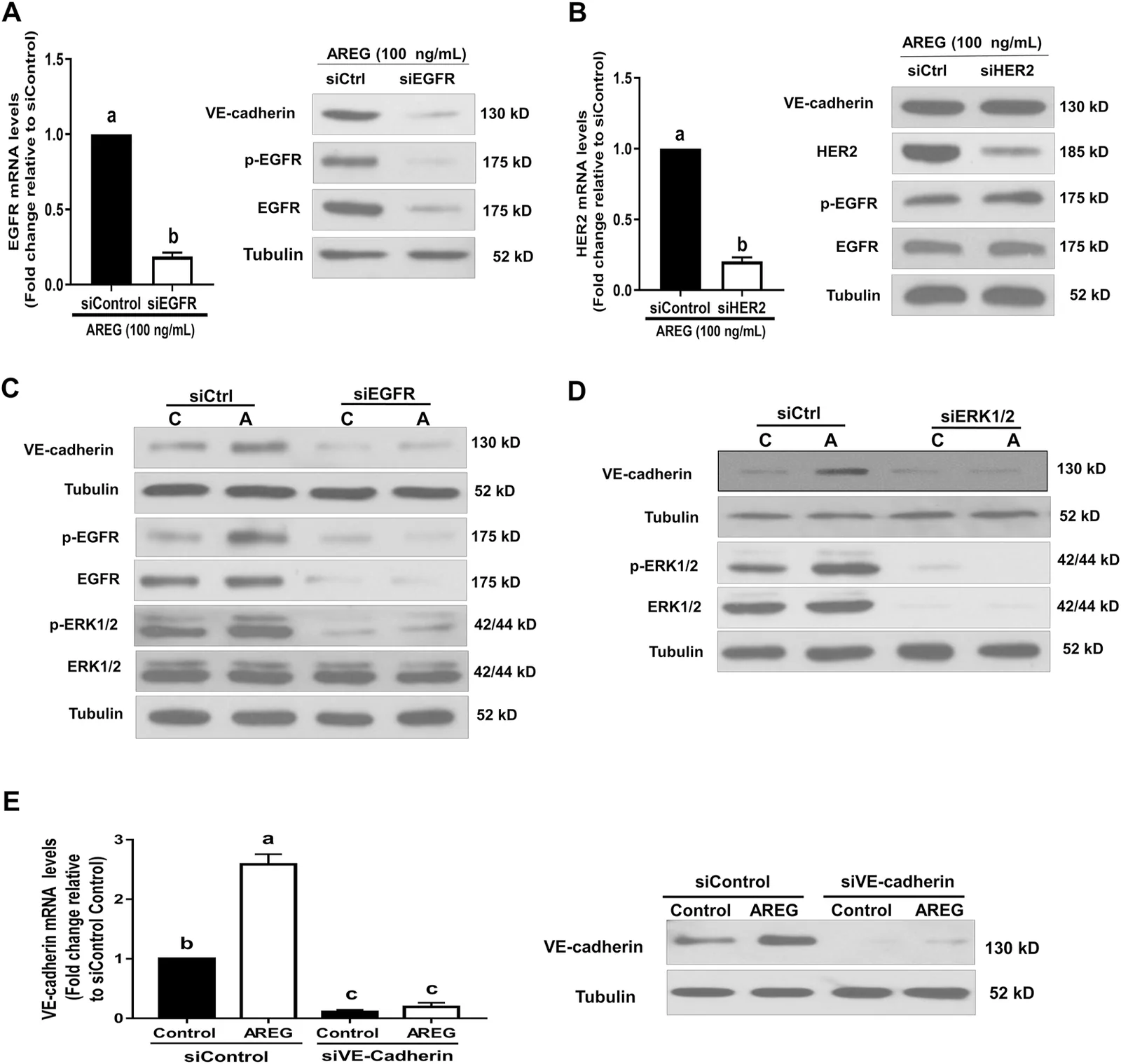
Effects of targeted depletion of EGFR, HER2, ERK1/2 and VE-cadherin on the AREG-induced upregulation of VE-cadherin. **(A,B)** SVOG cells were transfected with 25 nM siControl, siEGFR **(A)** or siHER2 **(B)** for 24 h in the presence of AREG (100 ng/ml), the expression levels of EGFR or HER2 were examined by RT-qPCR and western blotting, respectively. **(C,D)** Cells were transfected with 25 nM siControl, siEGFR **(C)** or siERK1/2 **(D)** for 24 h and then treated for an additional 15 min (for detecting phosphorylated EGFR and ERK1/2) or 24 h (for detecting VE-cadherin) with or without AREG (100 ng/ml). The phosphorylation levels of EGFR, ERK1/2 or VE-cadherin were examined by western blotting. **(E)** SVOG cells were transfected with 25 nM siControl or siVE-cadherin for 24 h and then treated for further 24 h with or without AREG (100 ng/ml). The expression levels of VE-cadherin were examined by RT-qPCR and western blotting, respectively. Values labeled with different letters are significantly different (*p* < 0.05) *n* = 3.

To avoid possible nonspecific effects of pharmacological inhibition, we used specific siRNA targeting EGFR and ERK1/2 to reconfirm the involvement of EGFR and ERK1/2 in AREG-induced upregulation of VE-cadherin expression. After transfection with EGFR-specific siRNA for 24 h, EGFR expression levels (both protein and mRNA) significantly decreased (Figure 6A). However, transfection with HER2 specific siRNA for 24 h did not cause such an effect (Figure 6B). Notably, siRNA-mediated EGFR knockdown abolished AREG-induced VE-cadherin expression upregulation and ERK1/2 phosphorylation in SVOG cells (Figure 6C). Interestingly, the basal levels of VE-cadherin and phosphorylated ERK1/2 were decreased following knockdown of EGFR (for 24 h) (Figure 6C). Additionally, siRNA-mediated ERK1/2 knockdown (for 24 h) abolished AREG-induced upregulation of VE-cadherin expression (Figure 6D). Moreover, the basal protein levels of VE-cadherin decreased after ERK1/2 knockdown (for 24 h) (Figure 6D). Collectively, these results suggest that AREG upregulates the upregulation of VE-cadherin expression in hGL cells *via* the EGFR-ERK1/2 signaling pathway.

### Epidermal growth factor receptor-ERK1/2 signaling-mediated upregulation of VE-cadherin expression contributes to amphiregulin-induced microvascular-like formation in SVOG cells

**FIGURE 7:**
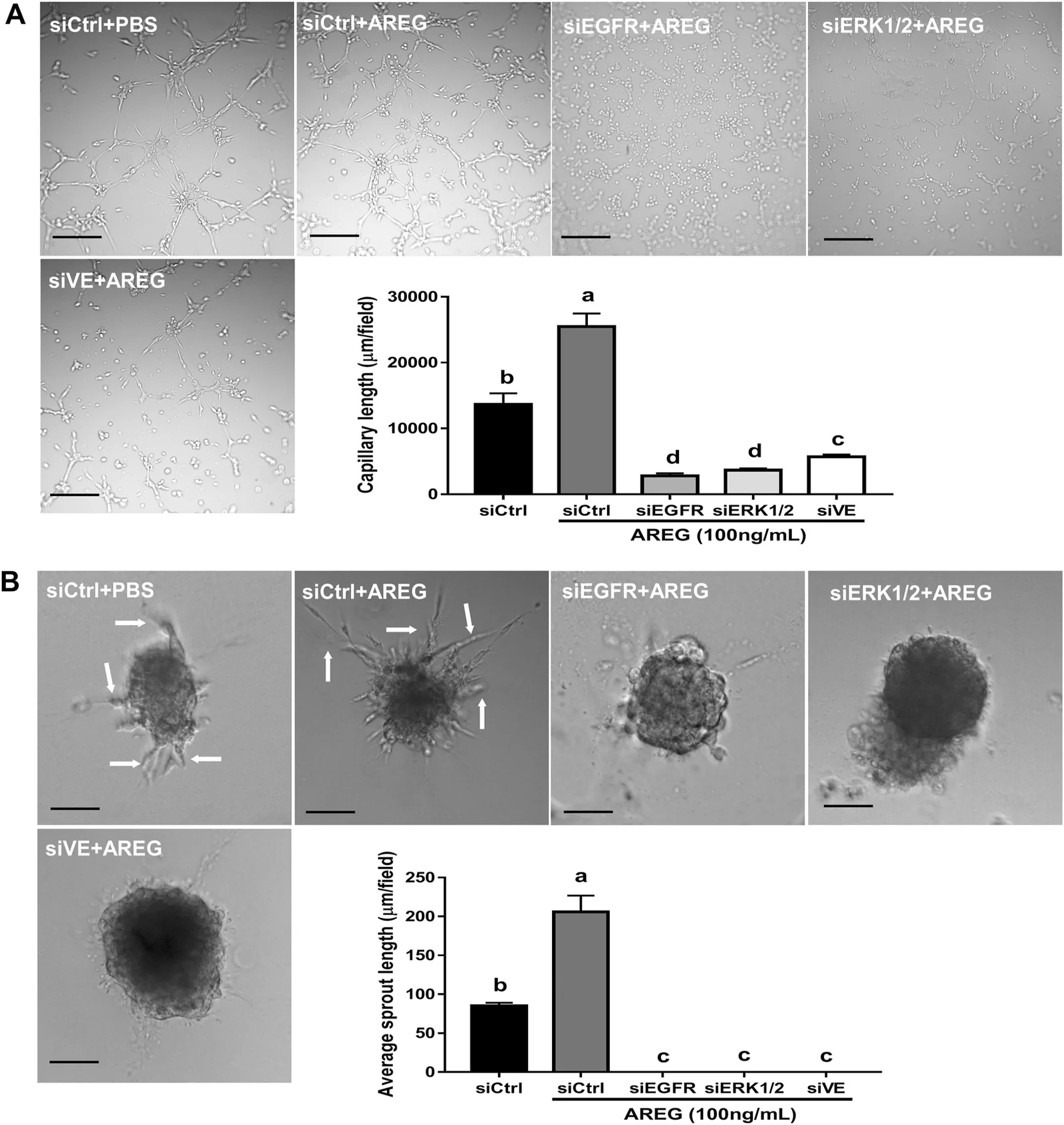
Effects of targeted depletion of EGFR, ERK1/2 and VE-cadherin on AREG-induced microvasculature-like formation in SVOG cells. Cells were transfected with 25 nM siControl, siEGFR, siERK1/2 or siVE-cadherin for 24 h and treated for further 24 h with or without AREG (100 ng/ml). Finally, the cells were transferred to Matrigel for an additional 5 h (for capillary-like formation) **(A)** or 24 h (for sprout-like formation) **(B)**. Scale bars of the capillary-like formation and sprout-like formation represent 100 µm and 50 μm, respectively. The lengths of the capillaries and sprouts were quantified by ImageJ. Values labeled with different letters are significantly different (*p* < 0.05), *n* = 3.

Transient siRNA knockdown was carried out to further reveal the molecular mechanisms underlying AREG-induced microvascular-like formation in hGL cells. The VE-cadherin knockdown efficiency was confirmed at the mRNA and protein levels, respectively. Results showed that VE-cadherin expression levels were dramatically decreased (in both protein and mRNA levels) in SVOG cells after siRNA knockdown for 24 h (Figure 6E). Notably, siRNA-mediated EGFR, ERK1/2 or VE-cadherin knockdown (for 24 h) abolished the stimulatory effects of AREG on capillary-like and sprout-like formations in SVOG cells (Figures 7A,B). These results indicate that EGFR-ERK1/2-mediated upregulation of VE-cadherin expression is crucial for AREG-induced microvasculature-like formation in hGL cells.

### Canonical ALK5-SMAD2/3/4 signaling pathway engages in transforming growth factor β1-induced downregulation of VE-cadherin expression

**FIGURE 8:**
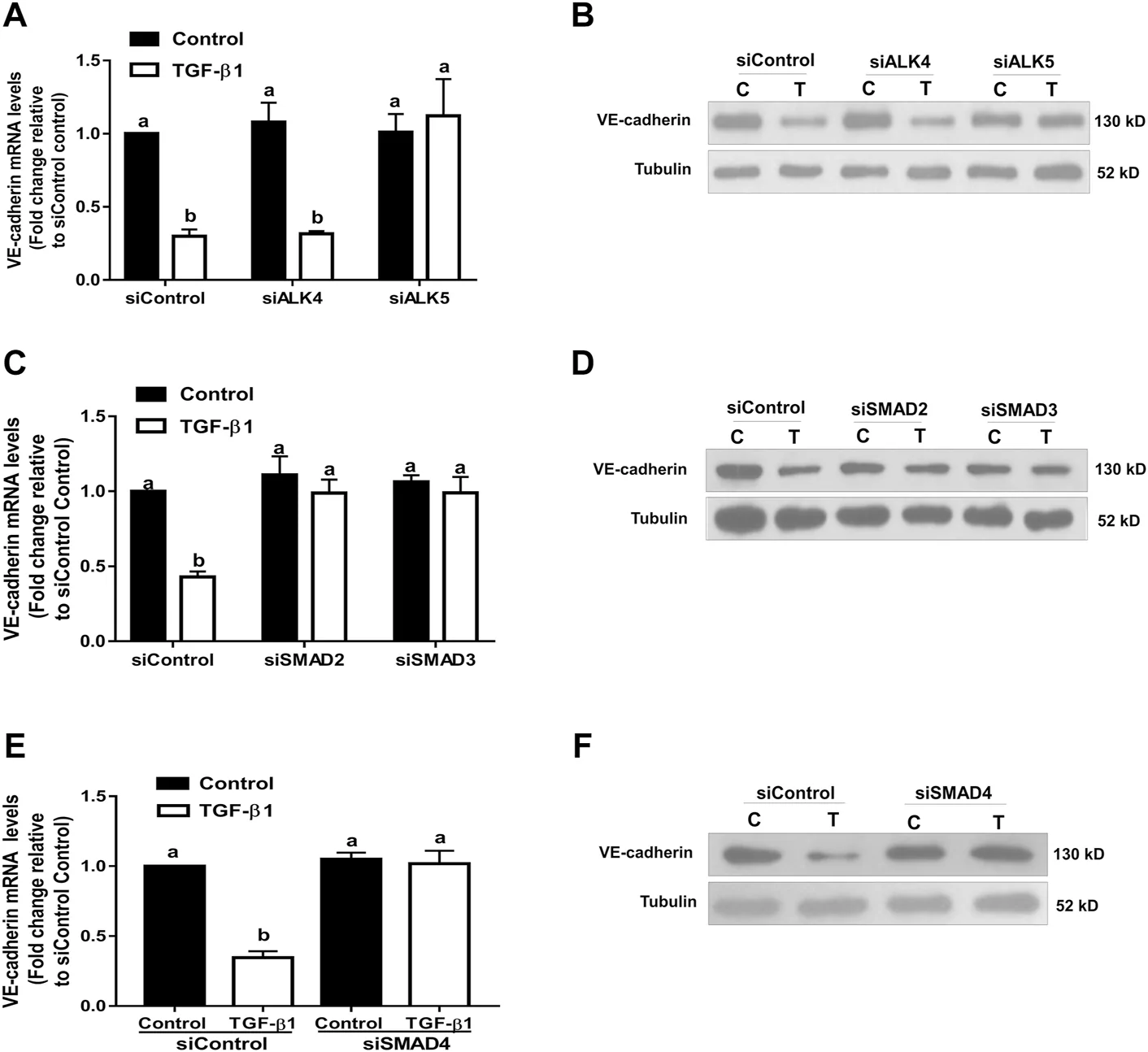
Effects of targeted depletion of ALK4, ALK5, SMAD2, SMAD3 and SMAD4 on the TGF-β1-induced VE-cadherin downregulation in SVOG cells. Cells were transfected with 25 nM siControl, siALK4, siALK5 **(A,B)**, siSMAD2, siSMAD3 **(C,D)** or siSMAD4 **(E,F)** for 24 h and treated for further 24 h with a vehicle control or TGF-β1 (10 ng/ml). The expression levels of VE-cadherin were examined by RT-qPCR and western blotting, respectively. Values labeled with different letters are significantly different (*p* < 0.05), *n* = 3. **(C)** control; T, TGF-β1.

**FIGURE 9:**
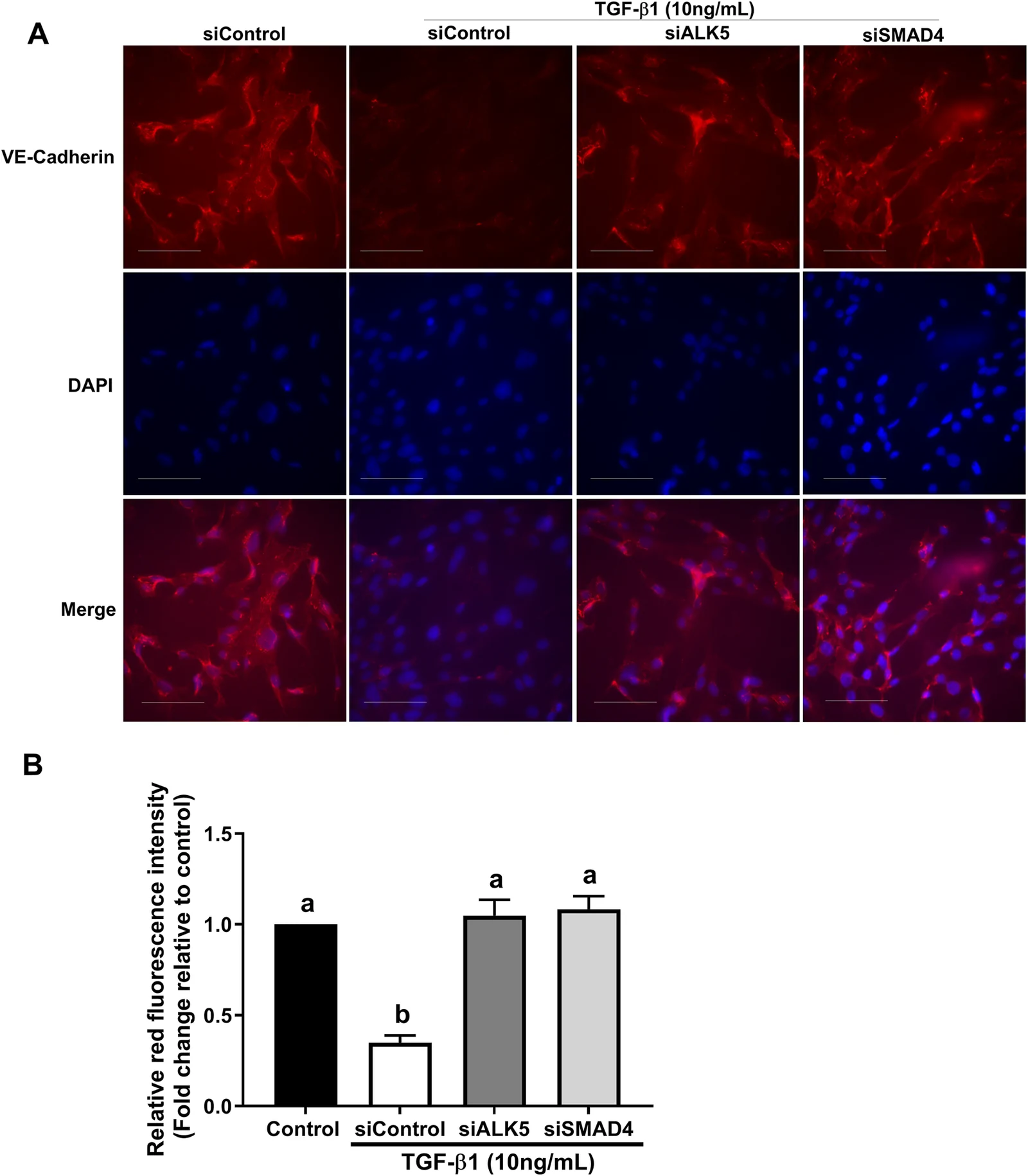
Fluorescence intensity assay showing the effects of targeted depletion of ALK5 and SMAD4 on the TGF-β1-induced VE-cadherin downregulation in SVOG cells. **(A)** Cells were transfected with siControl, siALK5 or siSMAD4 for 24 h and treated for further 24 h with a vehicle control or TGF-β1 (10 ng/ml). The VE-cadherin expression was determined using immunofluorescent staining. **(B)** The fluorescence intensity of VE-cadherin was further quantified by ImageJ. Scale bars represent 100 µm. Different letters above are represent significantly different (*p* < 0.05), *n* = 3.

Considering that the canonical ALK5-SMAD2/3/4 signaling pathway mediates several cellular functions in response to TGF-β1 treatment in hGL cells (Raffo-Romero et al., 2019), we performed transient siRNA-mediated knockdown to investigate the functional receptor and downstream signaling involved in TGF-β1-induced downregulation of VE-cadherin expression. Specifically, siRNA-mediated ALK5 knockdown (for 24 h) reversed the suppressive effect of TGF-β1 on VE-cadherin expression in SVOG cells (Figures 8A,B). Additionally, siRNA-mediated SMAD2, SMAD3 or SMAD4 knockdown (for 24 h) abolished TGF-β1-induced VE-cadherin decrease in SVOG cells (Figures 8C–F). Moreover, fluorescent staining experiments using an antibody against VE-cadherin showed that ALK5- or SMAD4-knockdown (for 24 h) significantly abolished the TGF-β1-induced fluorescence decrease in intensity SVOG cells (Figures 9A,B). Collectively, these results indicate that the canonical ALK5-SMAD2/3/4 signaling pathway mediates TGF-β1-induced VE-cadherin downregulation in hGL cells.

### The ALK5-SMAD/4 signaling-mediated downregulation of VE-cadherin contributes to transforming growth factor β1-induced suppression of microvasculature-like formation in SVOG cells

**FIGURE 10:**
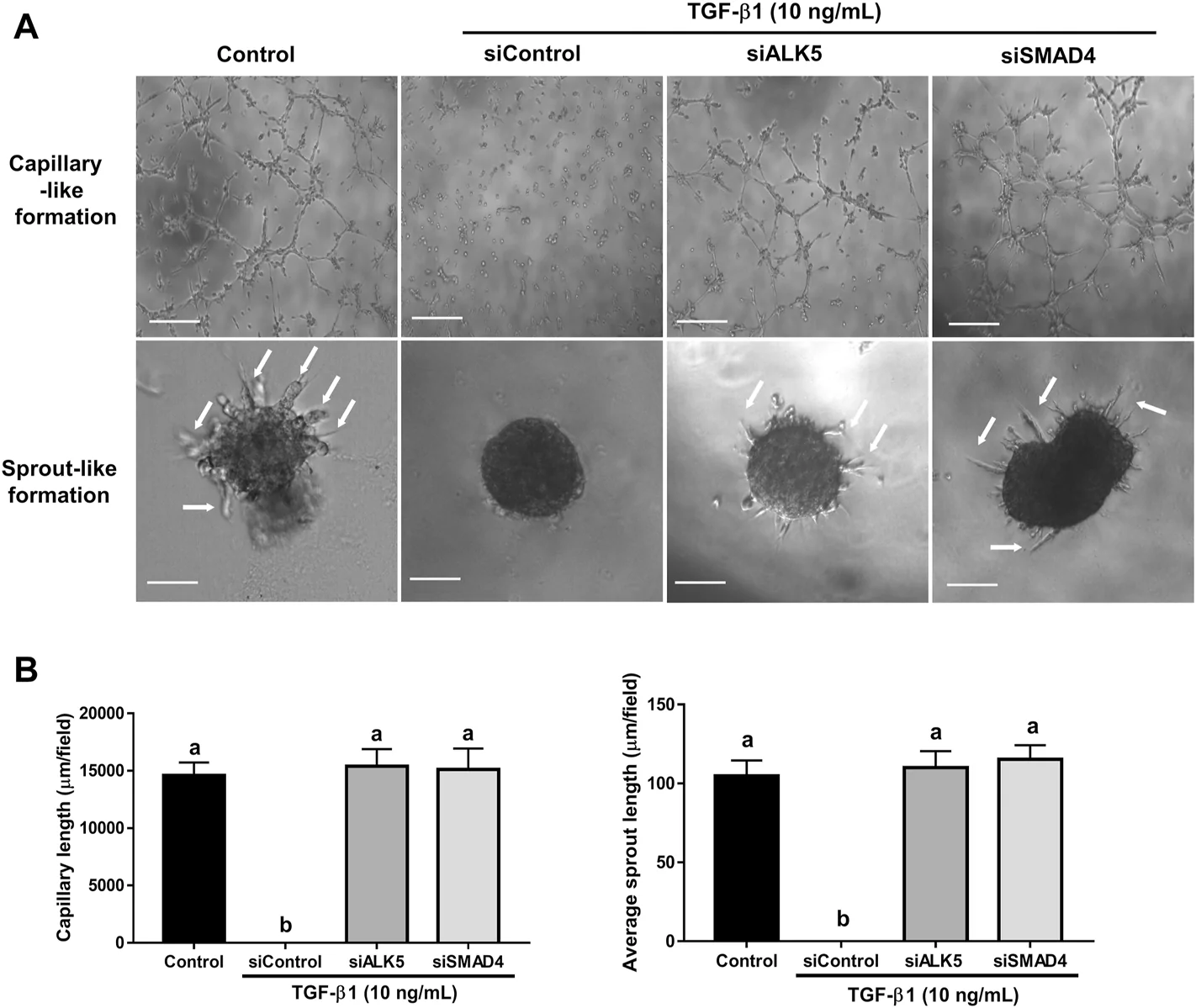
Effects of targeted depletion of ALK5 and SMAD4 on the TGF-β1-induced disturbance in microvasculature-like formation in SVOG cells. **(A)** Cells were transfected with siControl, siALK5 or siSMAD4 for 24 h and treated for further 24 h with or without TGF-β1 (10 ng/ml). Finally, cells were transferred to Matrigel for an additional 5 h (for capillary-like formation) and 24 h (for sprout-like formation). Scale bars of the capillary-like formation and sprout-like formation represent 100 µm and 50 μm, respectively. **(B)** The lengths of capillaries and sprouts were quantified by ImageJ. Different letters above are represent significantly different (*p* < 0.05), *n* = 3.

Finally, siRNA knockdown was performed to investigate the molecular mechanism underlying TGF-β1-induced disturbance of microvasculature-like formation in hGL cells. Notably, ALK5 or SMAD4 knockdown (for 24 h) reversed the adverse effects of TGF-β1 on capillary-like and sprout-like formation in SVOG cells (Figures 10A,B). These data indicate that the ALK5-SMAD4 signaling-mediated downregulation of VE-cadherin contributes to the TGF-β1-induced disturbance of microvasculature-like formation in hGL cells.

## Discussion

**FIGURE 11:**
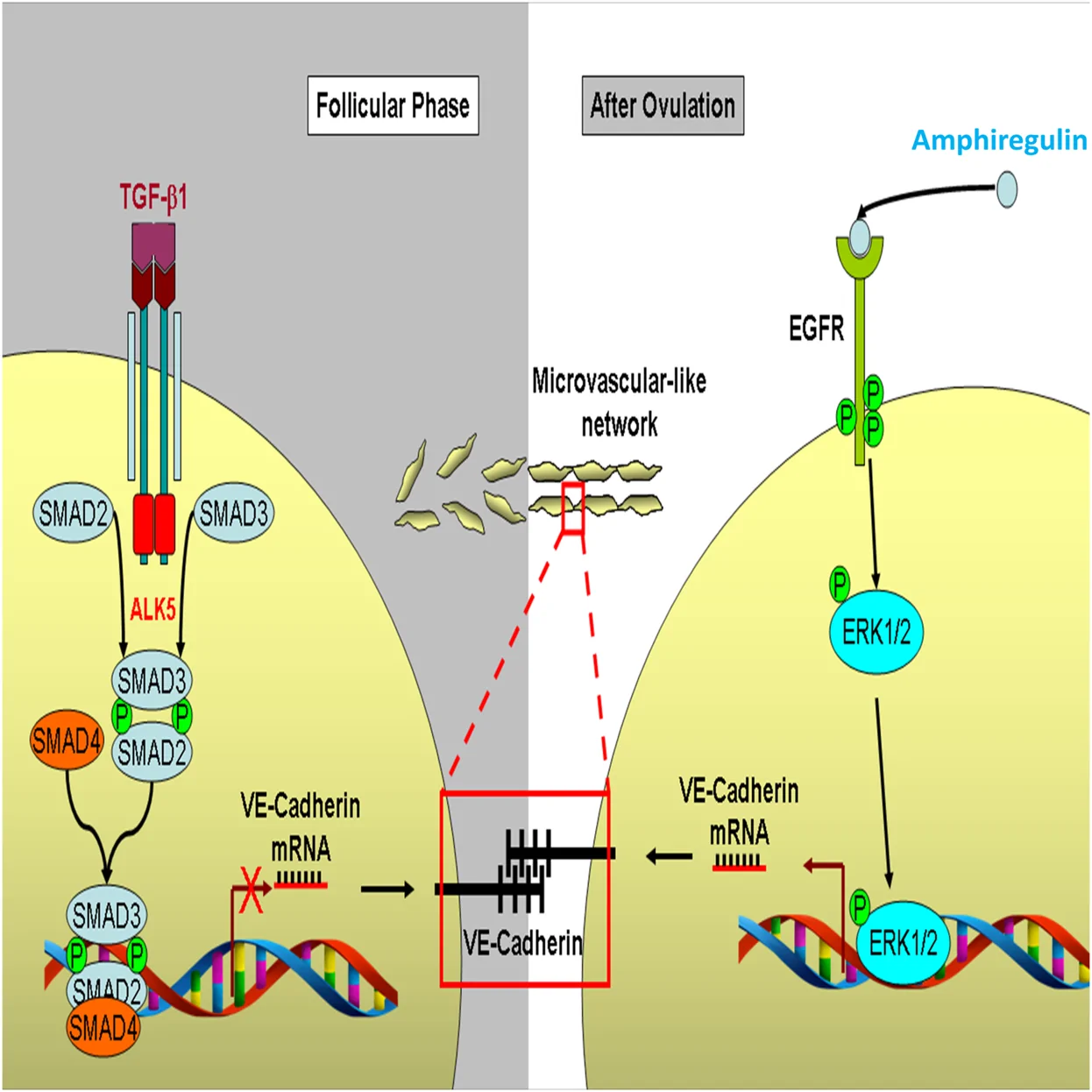
A proposed schematic diagram for the regulation of VE-cadherin and microvasculature-like formation in response to AREG and TGF-β1 in human granulosa cells. During the follicle phase, TGF-β1 binds to ALK5 and type II receptors in human granulosa cells which subsequently phosphorylates SMAD2/3, then combine with SMAD4, which in turn suppresses the transcription of VE-cadherin. The suppression of VE-cadherin inhibits the formation of intercellular microvascular-like networks. In contrast, after ovulation, human granulosa cells are luteinized to produce amphiregulin induced by LH. Amphiregulin binds to EGFR in an autocrine or paracrine manner and phosphorylates its downstream molecule ERK1/2 and subsequently promotes VE-cadherin expression, which finally promotes the formation of an intercellular microvasculature-like network during corpus luteum formation.

The corpus luteum is a transient endocrine gland that consists of luteinized GCs, TCs, and extraneous endothelial cells that develops in response to LH signaling (Rodger et al., 1997). The development of the corpus luteum is a complex event that involves an extensive and rapid vascularization transformation (Ratcliffe et al., 1999). In this study, the functional roles of two intrafollicular growth factors in regulating microvasculature-like structural changes in hGL cells were characterized, which represents one of the phenotypes of the human corpus luteum. Specifically, we showed that AREG promotes while TGF-β1 suppresses capillary-like and sprout-like formation in hGL cells. Previous studies in our lab have established that TGF-β1 acts as a potent luteal inhibitor by decreasing progesterone synthesis in hGL cells (Fang et al., 2014). However, LH surge-induced AREG promotes corpus luteum formation by increasing progesterone production in hGL cells (Fang et al., 2016). Our findings suggest that these two intraovarian regulators (AREG and TGF-β1) play opposite roles in regulating luteal function (Figure 11).

Matrigel is a mixture of membrane proteins containing extracellular matrix (ECM) components (Zhou et al., 2021). Different from other culture substrates, Matrigel can form a microenvironment that facilitates the differentiation of a wide variety of epithelial cells (Sodunke et al., 2007). For example, Matrigel decreases tissue fibrosis ratio, promotes angiogenesis and GC proliferation in mice with premature ovarian failure (POF) (Zhou et al., 2021). Additionally, Matrigel can promote *in vivo* angiogenesis (Passaniti et al., 1992; Ou et al., 2011) and *in vitro* microvasculature-like formation in endothelial cells (Wustehube et al., 2010). Collectively, the Matrigel culture system has been widely applied for mimicking the *in vivo* environment, especially for *in vitro* microvascular-like formation assay. Endothelial cell migration and hyperplasia are crucial cellular activities that facilitate vascularization during luteinization after ovulation (Peter and Leung, 2004). Moreover, several studies have shown that ovarian follicular GCs exhibit some overlapping functional features of the endothelial cells; they can form tube-like structures (characteristics of endothelial cells) by expressing several endothelial-specific cell markers (such as Tie, Tek, c-Kit, CD31 and vWF) (Antczak and Van Blerkom, 2000). Thus, GCs may potentially transform into vascularization structures or at least facilitate angiogenesis during corpus luteum formation. To further investigate the functions of GCs in vascularization activity, we cultured the SOVG and primary hGL cells onto Matrigel and found that these cells can spontaneously form capillary- and sprout-like networks, similar to those form by endothelial cells. Consistent with our findings, a previous study using a specific endothelial cell culture medium (EBM-2) showed that swine GCs exhibit similar phenotypic and functional characteristics (follicular angiogenesis, a crucial process in follicular growth and selection) to endothelial cells (Basini et al., 2016). Similarly, treatment of swine GCs with platelets shifts steroid production from estrogen to progesterone, which stimulates angiogenesis, indicating that platelets are directly involved in the luteinization of GCs (Mohamed et al., 2018).

In this study, we found that EGF receptors and VE-cadherin are highly expressed in hGL cells. VE-cadherin is another strictly endothelial-specific marker that engages in adherence junctions by linking with the extracellular domain of two VE-cadherin molecules between adjacent endothelial cells, while their cytoplasmic domain is anchored to the cytoskeleton (Nose et al., 1988; Takeichi, 1991). In this regard, VE-cadherin is an essential molecule involved in angiogenesis during embryonic development (Vestweber, 2008), cancer progression (Li et al., 2017; Han et al., 2018), wound healing (Cao and Schnittler, 2019) and corpus luteum formation (Nakhuda et al., 2005; Maroni and Davis, 2011). During angiogenesis, cells that form vessels must maintain stable cell–cell contacts; therefore, VE-cadherin mediates these intercellular contacts by stabilizing the endothelial structure (Etienne-Manneville, 2020). Indeed, studies have shown that VE-cadherin expression is downregulated in disorganized cells of angiosarcomas (Martin-Padura et al., 1995). Targeted depletion of VE-cadherin in mice leads to embryonic death because of severely disrupted vascular integrity (Gory-Faure et al., 1999). In this study, we found that VE-cadherin is the major molecule in hGL cells that mediates such microvascular-like structural changes in response to AREG and TGF-β1 treatment. In particular, AREG promoted microvascular-like formation by upregulating VE-cadherin expression; whereas, TGF-β1 suppressed microvascular-like formation by downregulating VE-cadherin expression. Indeed, the results of siRNA-mediated targeted depletion experiments demonstrated that knockdown of VE-cadherin completely reversed the effects of these two growth factors on capillary-like and sprout-like formation in hGL cells. Thus, VE-cadherin is essential in regulating vascularization, and AREG and TGF-β1 exert opposite regulatory functions in the expression of VE-cadherin and vascularization during corpus luteum development.

AREG is the most abundant protein produced in GCs and is highly expressed in human follicular fluid (Zamah et al., 2010; Fang et al., 2019). EGFR is the functional receptor that mediates AREG-induced cellular activity. Upon ligand binding, AREG activates EGFR activity and promotes several intracellular signaling pathways, including extracellular ERK1/2, PI3K/AKT/mTOR, p38MAPK and JAK/STAT (Rawlings et al., 2004; Oda et al., 2005; Engelman, 2009). In mouse ovaries, PI3K signaling is responsible for hCG-induced meiotic resumption, whereas ERK1/2 signaling predominantly mediates the ovulatory stimulus in periovulatory follicle (Shimada et al., 2003; Fan et al., 2009; Shimada et al., 2016). In the present study, we used an EGFR inhibitor (AG1478) to demonstrate that AREG-induced phosphorylation of EGFR and ERK1/2 and upregulation of VE-cadherin are mediated by EGFR. We further confirmed using siRNA-mediated knockdown of EGFR and ERK1/2 that AREG-induced upregulation of VE-cadherin expression is mediated by the EGFR-ERK1/2 signaling pathway. Therefore, the downstream signaling involved in the AREG-EGFR binding complex may be context or species-dependent.

Upon binding to type II receptor, TGF-β1 subsequently activates type I receptor (Massague, 2012). Using siRNA knockdown, we showed that ALK5 is the key molecule in TGF-β1-induced downregulation of VE-cadherin in hGL cells. Similarly, knockdown of SMAD2/3/4 reversed TGF-β1-induced downregulation of VE-cadherin. These results suggest that TGF-β1-induced VE-cadherin downregulation is mediated by the canonical ALK5-SMAD2/3/4 signaling pathway. Most importantly, siRNA knockdown of VE-cadherin reversed TGF-β1-induced suppressive effects on capillary-like and sprout-like formation, indicating that TGF-β1 inhibits vascularization during corpus luteum formation by suppressing VE-cadherin expression. Consistent with these results, TGF-β1 has also been shown to disturb the angiogenic potential in the corpus luteum (Maroni and Davis, 2011). However, TGF-β1 promotes angiogenesis in breast cancer by increasing VEGF expression (Petersen et al., 2010). These findings suggest that the regulatory effects of TGF-β1 on angiogenesis are cell type-dependent.

In summary, we have shown that AREG promotes the formation of microvascular-like networks in hGL cells by increasing VE-cadherin expression *via* the EGFR-ERK1/2 signaling pathway (Figure 11). By contrast, TGF-β1 suppresses microvascular-like network formation in hGL cells by downregulating VE-cadherin expression *via* the canonical ALK5-SMAD2/3/4 signaling pathway (Figure 11). Collectively, the present findings provide important insights into the mechanisms by which TGF-β1 and AREG differentially regulate corpus luteum formation in human ovaries.

## Data Availability

The original contributions presented in the study are included in the article/supplementary material, further inquiries can be directed to the corresponding authors.
