# Correction: Characterization of the roles of amphiregulin and transforming growth factor β1 in microvasculature-like formation in human granulosa-lutein cells

**DOI:** 10.3389/fcell.2026.1977869

**Published:** 2026-09-17

**Authors:** Hui Li, Hsun-Ming Chang, Saijiao Li, Christian Klausen, Zhendan Shi, Peter C. K. Leung

**Affiliations:** 1 Jiangsu Key Laboratory for Food Quality and Safety-State Key Laboratory Cultivation Base of Ministry of Science and Technology, Key Laboratory of Animal Breeding and Reproduction, Institute of Animal Science, Jiangsu Academy of Agricultural Sciences, Nanjing, China; 2 Department of Obstetrics and Gynaecology, BC Children’s Hospital Research Institute, University of British Columbia, Vancouver, BC, Canada; 3 Reproductive Medicine Center, Department of Obstetrics and Gynecology, China Medical University Hospital, Taichung, Taiwan; 4 Reproductive Medical Center, Renmin Hospital of Wuhan University, Wuhan, China

**Keywords:** VE-cadherin, TGF-β1, amphiregulin, microvasculature-like formation, human, granulosa cells

There was a mistake in Figure 9 as published. During figure assembly, the representative immunofluorescence images for the TGF-β1 + siSMAD4 group were inadvertently duplicated from Figure 5A and inserted into Figure 9A. The corrected Figure 9 appears below.

**FIGURE 9:**
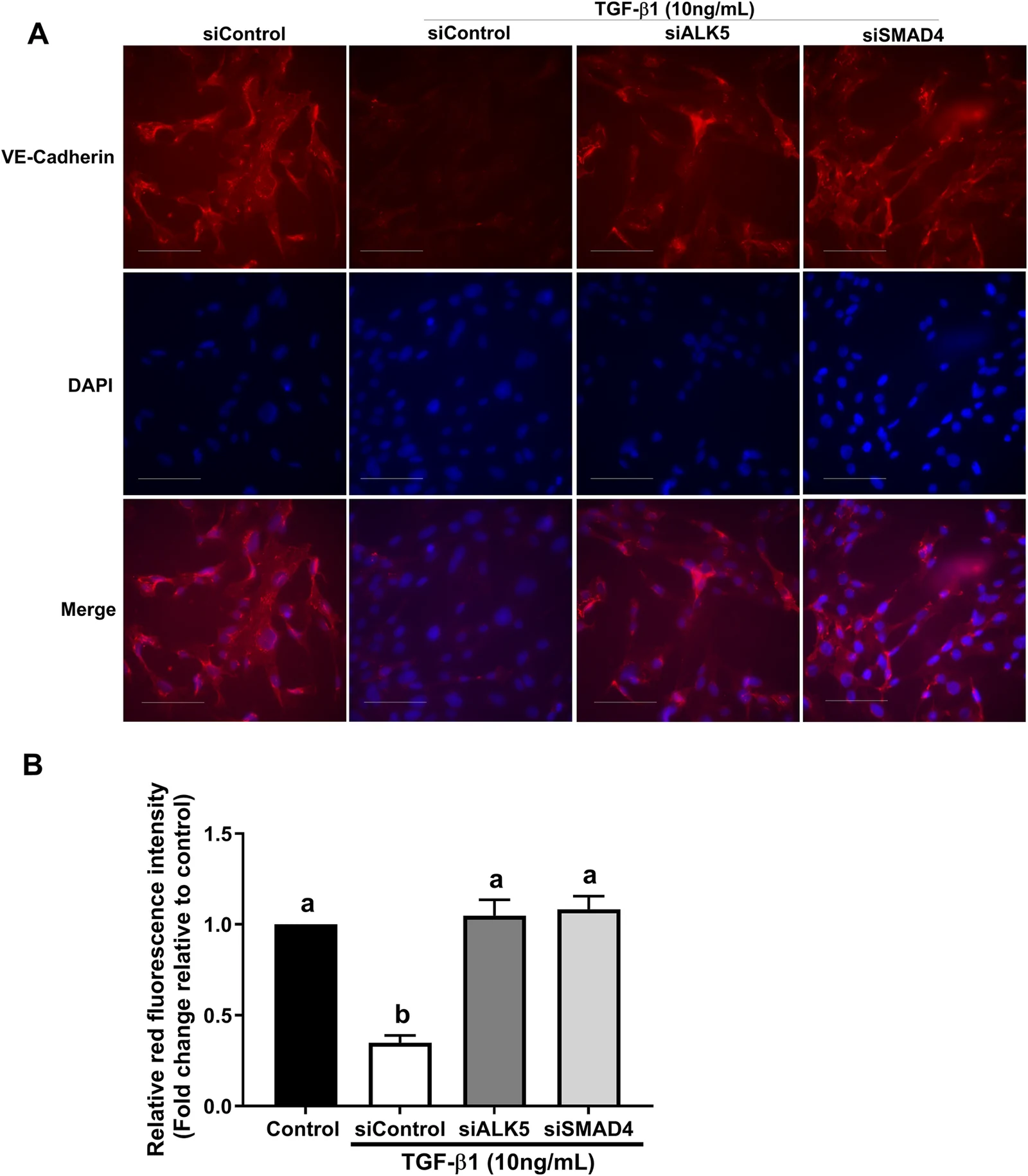
Fluorescence intensity assay showing the effects of targeted depletion of ALK5 and SMAD4 on the TGF-β1-induced VE-cadherin downregulation in SVOG cells. **(A)** Cells were transfected with siControl, siALK5 or siSMAD4 for 24 h and treated for further 24 h with a vehicle control or TGF-β1 (10 ng/mL). The VE-cadherin expression was determined using immunofluorescent staining. **(B)** The fluorescence intensity of VE-cadherin was further quantified by ImageJ. Scale bars represent 100 µm. Different letters above are represent significantly different (*p* < 0.05), *n* = 3.

The original version of this article has been updated.

